# Spontaneous Generation

**Published:** 1868-02

**Authors:** 


*Spontaneous Generation.*—Some months since we gave an account of the state of the controversy on this subject. It will be remembered that the distinguished microscopist, M. Donne, had gone over to the ranks of the spontaneous generationists, resting his convictions upon the results of certain experiments on eggs which he regarded as conclusive. We have now to announce that he has revised these experiments and has abandoned his new allies. He has been unable by the highest powers of his mi-microscope, to detect the smallest living organism in his prepared eggs. This greatly weakens the advocates of spontaneous generation, as his was the most distinguished name they could count on their lists. As we said before, the theory in question is contrary to the analogy of nature. It would require an overwhelming mass of proof to support it, and until that shall be brought to bear upon it, the scientific world will be slow to adopt the hypothesis.

